# Implementation of an institutional protocol for rational use of blood products and its impact on postoperative cardiac surgery

**DOI:** 10.1186/cc12674

**Published:** 2013-06-19

**Authors:** PGM de Barros e Silva, AC do Amaral Baruzzi, JT Garcia, MJ Rodrigues, MA Mieza, N Lasta, V Furlan, VA Fernandes

**Affiliations:** 1 Hospital Totalcor, Cerqueira César, São Paulo, SP, Brazil

## Introduction

Cardiac surgeries are sometimes followed by significant blood loss and transfusions may be necessary. However, indiscriminate use of blood components can result in detrimental effects for the patient. In this study, we evaluated the short-term effects of the implementation of a protocol for the rational use of blood products in the postoperative period of cardiac surgery.

## Methods

Between April and June 2011 an institutional protocol was implemented in a private hospital specialized in cardiology to encourage rational use of blood products with the consent and collaboration of seven cardiac surgery teams. Clinical and demographic data of patients were collected, and the use of blood products and clinical outcomes during in-hospital period 6 months before and after implementation of the protocol were analyzed. The protocol consisted of an institutional campaign with educational intervention in the surgical, intensive care and anesthesiology teams aiming to spread the practice of blood transfusion based on clinical goals (anemia with hemodynamic changes, significant ventricular dysfunction), as well as making routine prescription of epsilon aminocaproic acid (EACA) intraoperatively. Comparisons between categorical variables were performed with the chi-square test and *P <*0.05 was considered statistically significant.

## Results

After 3 months of implementation of the protocol, the use of EACA rose from 31 to 100%. The surgeries requiring any blood transfusion were 67% before the implementation of the protocol, and 40% in the subsequent months of the same year after implantation (*P *<0.001). Clinical outcomes related to blood transfusion are presented in Table 1 separating both periods.

**Table 1:** 

	Group 1 (pre-protocol)	Group 2 (after the protocol)
Acute renal failure (%)	9	6
Infection (%)	18	19
Mortality (%)	3	3

## Conclusion

The rational use of blood products associated with infusion of ε-aminocaproic acid has the potential to reduce the number of blood transfusions in postoperative of cardiac surgery, which can impact the risk of complications.

